# A New Hybrid Forecasting Model Based on SW-LSTM and Wavelet Packet Decomposition: A Case Study of Oil Futures Prices

**DOI:** 10.1155/2021/7653091

**Published:** 2021-07-12

**Authors:** Jie Wang, Jun Wang

**Affiliations:** ^1^Department of Statistics, College of Science, North China University of Technology, Beijing 100144, China; ^2^School of Science, Beijing Jiaotong University, Beijing 100044, China

## Abstract

The crude oil futures prices forecasting is a significant research topic for the management of the energy futures market. In order to optimize the accuracy of energy futures prices prediction, a new hybrid model is established in this paper which combines wavelet packet decomposition (WPD) based on long short-term memory network (LSTM) with stochastic time effective weight (SW) function method (WPD-SW-LSTM). In the proposed framework, WPD is a signal processing method employed to decompose the original series into subseries with different frequencies and the SW-LSTM model is constructed based on random theory and the principle of LSTM network. To investigate the prediction performance of the new forecasting approach, SVM, BPNN, LSTM, WPD-BPNN, WPD-LSTM, CEEMDAN-LSTM, VMD-LSTM, and ST-GRU are considered as comparison models. Moreover, a new error measurement method (multiorder multiscale complexity invariant distance, MMCID) is improved to evaluate the forecasting results from different models, and the numerical results demonstrate that the high-accuracy forecast of oil futures prices is realized.

## 1. Introduction

Crude oil is a natural and nonrenewable resource that has an irreplaceable effect on the development of the global economy and international financial markets. Since oil is the main source of energy production, it is often considered the single important commodity in the world. The price fluctuations of crude oil may affect the economic situation, social stability, and even national security in the world [1]. Meanwhile, international crude oil price series are regarded as nonlinear and nonstationary time series. Hence, accurate forecasting of the crude oil price is a challenging task of energy market and has increasingly become an active research field.

In recent years, numerous methods for time series predictions have been proposed [2–13]. These methods can be classified into the following three categories: traditional econometric models, machine learning approaches and deep learning models. The autoregressive integrated moving average model (ARIMA) is a popular statistical model applied to time series prediction. Liu et al. [3] proposed two novel forecasting models based on ARIMA, which was employed to forecast two sections of actual wind speed series. Abdollahi and Ebrahimi [4] established a new composite model to predict Brent crude oil prices by integrating the adaptive neuro fuzzy inference system (ANFIS), autoregressive fractionally integrated moving average (ARFIMA), and Markov-switching models. However, the traditional econometric models have evident shortcomings. For instance, the time series data must be stable when these models are used for forecasting. It is difficult to capture the characters if the datasets are nonstationary. Therefore, the model is less effective when applied for time series forecasting during periods of sharp fluctuations [14]. With the development of artificial intelligence, machine learning models, such as support vector machine (SVM) and artificial neural networks (ANNs), have attracted a lot of attention because of the learning capabilities for nonlinear kernel mapping between input and output vectors. For instance, Huang et al. [7] explored the forecasting ability of SVM for financial movement direction and proposed a combining model based on SVM and classification methods. Ghiassi et al. [15] presented a dynamic neural network model for time series events prediction, and compared with the ARIMA model, the prediction results of the proposed model have higher accuracy. Liao and Wang [6] established an improved neural network, the stochastic time-effective neural network model, and analyzed the volatility statistics characteristics of the Chinese stock price indices. Wang and Wang [8] established a hybrid model by combining the principle component analysis (PCA) algorithm and random time-effective neural networks (STNN) and explored the predictive performance by considering financial time series. Although machine learning techniques have considerable prediction processing capacity, their precision on the correlations exploring between data is still not efficient. Meanwhile, these methods are extremely time-consuming for big data and predictions are not quite expected [16]. With the establishment of the hidden layer units, the transmission of historical information can be realized by recurrent neural networks (RNNs). Wang and Wang [9] proposed a new forecasting model to elevate the prediction accuracy of crude oil price fluctuations, which is based on multilayer perceptrons (MLP) and Elman recurrent neural networks (ERNN) with stochastic time effective function. Berradi and Lazaara [17] combined principal component analysis and RNNs to predict the stock price from Casablanca Stock Exchange, and the results enhanced the accuracy of the original method and performed a desirable prediction for the stock price. Deep learning methods are the broader series of machine learning methods, which try to learn advanced features from the given data. Compared with traditional neural network models, deep learning methods contain multiple hidden layers of multilayer perceptrons, and they have better performances in managing strong nonlinear characteristics. Long short-term memory network (LSTM) is a type of deep learning method devised to deal with the long-term dependence problems for a special purpose [18]. The network structure of LSTM is much more complex than that of RNNs, which utilizes memory cell states to maintain essential historical information and get rid of the unimportant. Due to the superior algorithm mechanism, LSTM is widely applied to natural language processing (NLP) and sentimental analysis [19, 20], time series forecasting [10, 21, 22], and synthesizing a piece of music [23]. However, the individual forecasting models cannot precisely reveal the complicated connections existing in the nonlinear and nonstationary datasets.

To obtain more accurate and reliable time series prediction, different kinds of hybrid forecasting models have been proposed which could take the advantage of different single models [24–26]. Among them, the hybrid models based on decomposition and prediction have been widely recognized, and such models are usually composed of nonlinear decomposition method and forecasting model. Liu et al. [27] presented an improved hybrid forecasting model for wind speed, which includes the empirical wavelet transform method and three types of deep learning networks. By comparing all the data results of different methods, the proposed reinforcement learning based hybrid model is effective in combining three types of deep learning networks and performs better than conventional optimization-based hybrid models. Wang and Wang [28] combined empirical mode decomposition (EMD) method with random time strength neural network to predict global stock indices, and the empirical results showed that the proposed approach veritably has a great effect in predicting stock market fluctuations. Wang et al. [29] established a two-layer decomposition model and then developed an ensemble approach by integrating the fast ensemble empirical mode decomposition method (FEEMD), variational mode decomposition (VMD), and optimized backpropagation neural network by firefly algorithm (FA-BPNN). The empirical results indicated that the developed new model has exceptional forecasting implementation in electricity price series. The first key point of hybrid models is to break down the original data series into several independent subseries and makes it likely for models to adaptively learn the nonlinear characteristics of fluctuations in each subseries. Then, by using the inverse transformation algorithm, the forecasting series of each subseries are integrated to acquire the final forecasting results. These hybrid models could raise the efficiency and precision of modelling by conquering the handicap of nonlinear and nonstationary of original series [30–32]. The empirical results show that wavelet transform (WT) is a time-frequency localization analysis method in which the window area is fixed but its shape can be changed. Because it only redecomposes low-frequency signals during the decomposition process, and no longer breaks down high-frequency signals, its frequency resolution decreases as the frequency increases. The EMD, FEEMD, and VMD methods also have some certain limitations, for example, inadequate mathematical explanations, the boundary effects, noise oversensitivity, and pattern overlap. These may cause excessive decomposition of the original data and adversely affect the prediction results [33, 34]. On the other hand, the well-known deep learning model causes overfitting problems and is always based on historical information without thinking over the statistical regularity of behavior in the financial market, which leads to deficient precision [10, 32].

To improve the disadvantages of the above widely recognized decomposition methods and the traditional deep learning methods, this paper proposes a novel ensemble energy forecasting framework, WPD-SW-LSTM, which combines wavelet packet decomposition (WPD), the stochastic time strength weights (SW) method, and LSTM. The WPD is proposed on the basis of the issue that the inferior frequency resolution of wavelet decomposition in the high-frequency range and poor time resolution in the low-frequency range. It is a more sophisticated method of signal analysis to improve the temporal resolution signal. Moreover, the WPD working speed is faster than the traditional WT, and by selecting the appropriate wavelet basis function and mother function, the mixing-frequency problem can be improved. Therefore, WPD is adopted in this research to explore the complexity of nonlinear characteristics for original energy future time series. In fact, there are complicated factors that affect energy futures prices in the process of market transactions fluctuations. SW is based on stochastic process which conforms with both the real trading market and the gating mechanism in the forecasting model [6, 8, 10]. The mechanism of SW is to measure historical information in conformity with the time of occurrence. The newer the historical data occurs, the more valuable its data information is to present future information, so that historical price figures can be employed to advanced pick up the fluctuations statistics in the energy futures series. In addition, this research employs the WPD method to extract the original crude oil series for the first time and firstly improves the conventional LSTM model with stochastic time strength weights for the crude oil prices forecasting. With the method of WPD, the original energy futures price series can be decomposed into several subseries (SS_*i*_), which are in different frequency bands. Then, different SW-LSTM models are modeled for the corresponding SS_*i*_, respectively. Finally, the ensemble forecasting result of the original energy futures series is produced by integrating all the predicted SS_*i*_ components. To estimate the predictive power of the proposed model WPD-SW-LSTM, the conventional and latest hybrid models (SVM, BPNN, LSTM, WPD-BPNN, WPD-LSTM, CEEMAD-LSTM, VMD-LSTM, and ST-GRU) are introduced for comparative analysis. In order to reveal the predictive capabilities of different forecasting models, quantitative analysis is performed through different error methods. At the same time, this research proposes a new error measurement method called multiorder multiscale complexity invariant distance (MMCID) [9,35]. The main contributions of this paper are summarized as follows:

- A novel hybrid forecasting model SW-LSTM is established for energy futures series, which based on the LSTM network and the theory of stochastic process.
- Combined with WPD method, several subseries (SS_*i*_) with different fluctuation frequency are derived from the original data series. Each SS_*i*_ is trained by the new SW-LSTM model, respectively.
- The empirical results of corresponding forecasting models are estimated and contrasted with different error criteria and the new measurement MMCID.

The structure of this article is as follows.  Section 2 explains the price datasets from the energy futures markets.  Section 3 introduces the WPD and SW-LSTM methodologies and provides the main framework of this paper.  Section 4 demonstrates the experimental forecasting results in detail.  Section 5 compares the proposed hybrid method with other models, which are SVM, BPNN, LSTM, WPD-BPNN, WPD-LSTM, CEEMAD-LSTM,VMD-LSTM, and ST-GRU. Moreover, error measurement methods are applied to estimate the prediction performance of each model in this section. Finally, Section 6 summarizes the main conclusion of this study.

## 2. Datasets

**Figure 1:**
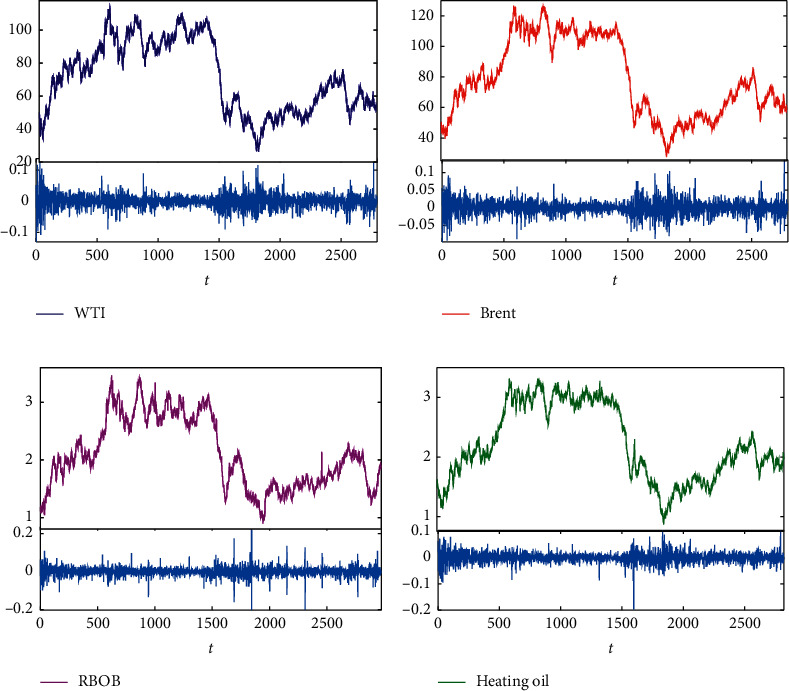
Dynamic changes of energy futures series between January 2, 2009, and October 23, 2019.

Crude oil is an international bulk financial commodity, which can be traded in markets around the world either through spot oil or through financial derivative contracts. This research mainly focuses on the oil futures market, and four representative oil futures indices are selected for the case study: west Texas intermediate (WTI) futures prices series, Brent crude oil futures prices series, RBOB gasoline, and heating oil. These four datasets are from the New York Mercantile Exchange (NYMEX) energy futures market, which can be downloaded from https://www.wind.com.cn/. WTI crude oil price is widely applied in the pricing of US domestic crudes. Brent is the theoretical international oil benchmark, and prices of most oil use Brent crude as the criterion, which connected with two-thirds of all the world's oil contracts. Brent crude and WTI dominate the oil market, and both determine pricing in their corresponding markets. They are known as light sweet oil because they contain low sulfur, making it “sweet,” and have low density, making it “light.” Gasoline and heating oil are refined from crude oil which are usually merchandised as futures contracts in financial markets.  Figure 1 reveals the similar dynamic changes in more than a 10-year period from January 2, 2009, to October 23, 2019, of the four corresponding oil futures series. In the past decades, the price fluctuation trends of these four futures series are almost the same, which manifest that there is a certain correlation between them.

## 3. Methodology

### 3.1. Wavelet Packet Decomposition

Wavelet transform is a mathematical method produced to solve the problem of decomposition of nonstationary signals. Compared with wavelet analysis, wavelet packet decomposition (WPD) can be used to analyze the signal more meticulous. Wavelet packet analysis can divide the time-frequency plane in more detail, and the resolution of the high-frequency part of the signal is better than wavelet analysis [36]. It can also adaptively select the best wavelet basis function according to the characteristics of the signal in order to better analyze the signal. The theory of the WPD analysis is as follows [37–39]. The wavelet packet function is a time-frequency function; it can be defined as

(1) $$\begin{array}{c} W_{j,k}^{n}\left(t\right)=2^{\left(j/2\right)}W^{n}\left(2^{j}t-k\right), \end{array}$$

where the integers *j* and *k* are the index scale and translation operations. The index *n* is an operation modulation parameter or oscillation parameter. The first two wavelet packet functions are the scaling and mother wavelet functions:

(2) $$\begin{array}{c} W_{0,0}^{0}\left(t\right)=\phi \left(t\right),\\ W_{0,0}^{1}\left(t\right)=\psi \left(t\right). \end{array}$$

When *n*=2,3,…, the function has the following recursive relationship:

(3) $$\begin{array}{c} W_{0,0}^{2n}\left(t\right)=\sqrt{2}\underset{k}{\sum }h\left(k\right)W_{1,k}^{n}\left(2t-k\right),\\ W_{0,0}^{2n+1}\left(t\right)=\sqrt{2}\underset{k}{\sum }g\left(k\right)W_{1,k}^{n}\left(2t-k\right), \end{array}$$

where *h*(*k*) and *g*(*k*) are the quadrature filter function related to the previously defined scaling function and mother wavelet function. The wavelet packet coefficients *w*_*j*,*k*_^*n*^ are calculated by the inner product 〈*f*(*t*)*W*_*j*,*k*_^*n*^〉, which is defined as

(4) $$\begin{array}{c} w_{j,k}^{n}=\langle f\left(t\right)W_{j,k}^{n}\rangle =\int f\left(t\right)W_{j,k}^{n}\text{d}t. \end{array}$$

**Figure 2:**
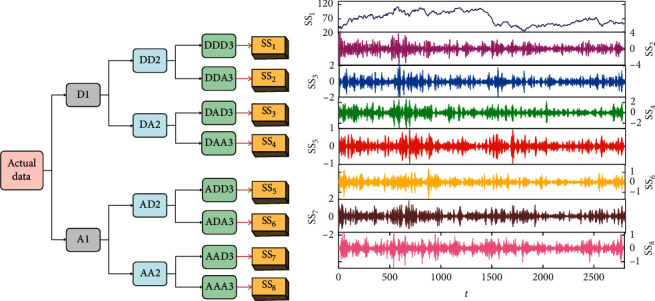
(a) The process of WPD algorithm. (b) The corresponding subseries SS_*i*_ of WTI index derived from WPD.

According to the literature [40], the number of the decomposition level is often in the range from 2 to 4 in forecasting model. In the present work, the 3-level framework of WPD algorithm is applied, which is schematically shown in Figure 1(a). Additionally, the Daubechies wavelets of order 4 are employed as the mother wavelet in this research [41], and the corresponding decomposition result of the WTI crude oil is demonstrated in Figure 2(b). Each subseries with different frequency band represents a sort of oscillatory factor embedded in the futures price indices. In Figure 2(b), the decomposed subseries “DDD3,” “DDA3,” “DAD3,” “DAA3,” “ADD3,” “ADA3,” “AAD3,” “AAA3” are recorded as SS_*i*_(*i*=1,2,…, 8) series subsequently.

### 3.2. Long Short-Term Memory Network

**Figure 3:**
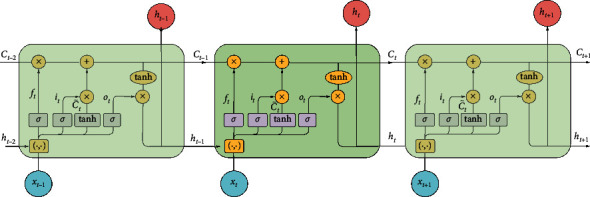
The architecture of LSTM network.

Long short-term memory networks are a particular form of RNNs that can handle with long-term and short-term dependencies. They were introduced in 1997 by Hochreiter and Schmidhuber [18] and were improved and promoted in subsequent work. Although the structure of traditional RNNs are entirely component of handling long-term memory dependencies in theory, the effect is confined in the actual application [42]. Therefore, the memory storage capacity of RNNs is more suitable for short-term sequences. On the basis of conventional RNNs, cell states and gate mechanism are added to the hidden layer, so that the gradient vanishing problem can be largely mitigated through its control gates. In addition, each time the historical message is dispatched to the neurons of the hidden layer, several control gates with different functions are employed to regulate the information of the past and latest. The principle of the control gate is described as follows. It is mainly composed of a sigmoid neural net layer and a pointwise multiplication operation. The output values of sigmoid function stage are between 0 and 1, which indicate how much information can be delivered to the next step. A value of zero means letting nothing through, while a value of one means letting everything through. Specially, when the value is 0, it means nothing can be transmitted, and when the value is 1, it implies everything can be transmitted. The LSTM control gates involve three gates: the forget gate *f*_*t*_, the input gate *i*_*t*_, and the output gate *o*_*t*_. The forget gate determines how much historical information stored in the current moment from the last moment. The input gate judges the information saved in the cell state, and the output gate decides the output data based on the cell state. The architecture of LSTM network is shown in Figure 3. The description of LSTM networks follows Fischer and Krauss [43], Sainath et al. [44], and He et al. [45]. The specific algorithm steps of LSTM are as follows:

(i) The memory cell reads in the input *x*_*t*_ and the previous hidden state *h*_*t*−1_, which can reveal long-term dynamic trends and abandon the redundant useless information. The forget gate is determined by the following equation:
  (5) $$\begin{array}{c} f_{t}=\sigma \left\{W_{f}\cdot \left(x_{t},h_{t-1}\right)+b_{f}\right\}. \end{array}$$
(ii) The first part of input gate in the model determines how much current information should be retained in the cell state:
  (6) $$\begin{array}{c} i_{t}=\sigma \left\{W_{i}\cdot \left(x_{t},h_{t-1}\right)+b_{i}\right\}. \end{array}$$
(iii) The second part is to generate a new candidate vector ${\tilde{C}}_{t}$ to update the state, which is according to the following equation:
  (7) $$\begin{array}{c} {\tilde{C}}_{t}=\tanh\left\{W_{C}\cdot \left(x_{t},h_{t-1}\right)+b_{C}\right\}. \end{array}$$
(iv) After that, the new cell state *C*_*t*_ is constructed on the basis of the outcomes of the last steps with ⊗ denoting the Hadamard (element-wise) product:
  (8) $$\begin{array}{c} C_{t}=f_{t}⊗C_{t-1}+i_{t}⊗{\tilde{C}}_{t}. \end{array}$$
(v) Finally, the output gate *o*_*t*_ is updated and the final output *h*_*t*_ is decided based on the updated state and the output gate state:

(9) $$\begin{array}{c} o_{t}=\sigma \left\{W_{o}\cdot \left(x_{t},h_{t-1}\right)+b_{o}\right\},\\ h_{t}=o_{t}⊗\;\tanh\left(C_{t-1}\right). \end{array}$$

In the previous equations, the following notation is used:

(i) *x*_*t*_ is the input vector at current time step *t*.
(ii) *W*_*f*_, *W*_*i*_, *W*_*C*_, and *W*_*o*_ are the weight matrices which associate with corresponding vectors. They can be spilt into
  (10) $$\begin{array}{c} \left\{\begin{array}{c} W_{f}=W_{fx}+W_{fh},\\ W_{i}=W_{ix}+W_{ih},\\ W_{C}=W_{Cx}+W_{Ch},\\ W_{o}=W_{ox}+W_{oh}. \end{array}\right) \end{array}$$
(iii) *b*_*f*_, *b*_*i*_, *b*_*C*_, and *b*_*o*_ are bias indicators.
(iv) *f*_*t*_, *i*_*t*_, and *o*_*t*_ are forget gate, input gate, and output gate vectors.
(v) *C*_*t*_ and ${\tilde{C}}_{t}$ are vectors for the cell states and candidate values.
(vi) *h*_*t*_ is a vector for the output of the LSTM layer.
(vii) *σ*(·) and tanh(·) are the sigmoid function and hyperbolic tangent function, respectively.

### 3.3. LSTM with Stochastic Time Effective Weight Function (SW-LSTM)

Dufresne and Gatheral et al. [46, 47] demonstrate that the prediction of financial market price series should integrate great amount of historical data, because the information represented in different periods has different impacts on future results. In other words, the closer the data is to the current time, the stronger the impact of information is at that moment, and, on the contrary, the further the data is, the weaker the influence is [48]. Therefore, to improve the accuracy of forecasting in actual application, this paper considers combining the SW function with LSTM theory in the predictive modelling process. During the stage of model training, SW function is integrated into the LSTM model to construct a novel forecasting model, which is referred to as long short-term memory with stochastic time strength weight function model (SW-LSTM). The expression of SW function derives from a stochastic process [6]. It can assign different weights to different data in the light of the variant time of occurrence. The mathematical expression is as follows:

(11) $$\begin{array}{c} \varphi \left(t_{n}\right)=\frac{1}{\beta }\exp\left\{\int_{t_{0}}^{t_{n}}\mu \left(t\right)\text{d}t+\int_{t_{0}}^{t_{n}}\omega \left(t\right)\text{d}B\left(t\right)\right\}, \end{array}$$

where *β*(>0) is the depth of market parameter, *t*_0_ is the moment of the latest time point in the data set, and *t*_*n*_ is an arbitrary time point in the dataset. *B*(*t*) is the standard Brownian motion which is commonly considered as random movement of a particle in liquid [49]. *μ*(*t*) is the drift function which mainly direct trend changes. *ω*(*t*) is the wave function which is applied to model the uncertain events during the forecasting process. The mathematical expression of *μ*(*t*) and *ω*(*t*) is as follows:

(12) $$\begin{array}{c} \mu \left(t\right)=\exp\left(-\alpha t\right),\\ \omega \left(t\right)=\omega \left(T\right)=\frac{1}{T-1}{\left(\sum_{i=1}^{T}{\left(x_{i}-\bar{x}\right)}^{2}\right)}^{\left(1/2\right)}. \end{array}$$

In the training process of conventional LSTM network, the parameter matrices *W*_*f*_, *W*_*i*_, *W*_*C*_, and *W*_*o*_ are modified following the backpropagation in each iteration through time procedure of typical RNNs [17]. The model training error of the sample point *n* is defined as

(13) $$\begin{array}{c} E\left(t_{n}\right)=\frac{1}{2}\;\varepsilon_{t_{n}}^{2}=\frac{1}{2}{\left(d_{t_{n}}-y_{t_{n}}\right)}^{2}. \end{array}$$

For the SW-LSTM model, a new description of model training error *E*_*tn*_ can be obtained:

(14) $$\begin{array}{c} E\left(t_{n}\right)=\frac{1}{2}\varphi \left(t\right)\varepsilon_{t_{n}}^{2}=\frac{1}{2}\varphi \left(t\right){\left(d_{t_{n}}-y_{t_{n}}\right)}^{2}. \end{array}$$

Then, the corresponding global error of model training is defined as

(15) $$\begin{array}{c} E=\frac{1}{N}\sum_{i=1}^{N}E_{t}=\frac{1}{2N}\sum_{i=1}^{N}\frac{1}{\beta }\exp\left\{\int_{t_{0}}^{t_{n}}\mu \left(t\right)\text{d}t+\int_{t_{0}}^{t_{n}}\omega \left(t\right)\text{d}B\left(t\right)\right\}{\left(d_{t_{n}}-y_{t_{n}}\right)}^{2}. \end{array}$$

In the modelling process, based on the newly defined global error *E*, the model parameters are updated through the gradient descent method [10, 50, 51]. First, the partial derivative of each model parameter needs to be calculated from the global error function. Then, the principle of parameter update is as follows:

(16) $$\begin{array}{c} \frac{\partial E}{\partial W_{fx}}=\frac{\partial E}{\partial {\text{net}}_{f,t}}\frac{\partial {\text{net}}_{f,t}}{\partial W_{fx}}=\delta_{f,t}\varphi \left(t\right)x_{t},\\ \frac{\partial E}{\partial W_{fh}}=\overset{t_{n}}{\underset{j=t_{0}}{\sum }}\delta_{f,j}\varphi \left(t\right)h_{j-1},\\ \frac{\partial E}{\partial W_{ix}}=\frac{\partial E}{\partial {\text{net}}_{i,t}}\frac{\partial {\text{net}}_{i,t}}{\partial W_{ix}}=\delta_{i,t}\varphi \left(t\right)x_{t},\\ \frac{\partial E}{\partial W_{ih}}=\overset{t_{n}}{\underset{j=t_{0}}{\sum }}\delta_{i,j}\varphi \left(t\right)h_{j-1},\\ \frac{\partial E}{\partial W_{Cx}}=\frac{\partial E}{\partial {\text{net}}_{C,t}}\frac{\partial {\text{net}}_{C,t}}{\partial W_{Cx}}=\delta_{C,t}\varphi \left(t\right)x_{t},\\ \frac{\partial E}{\partial W_{Ch}}=\overset{t_{n}}{\underset{j=t_{0}}{\sum }}\delta_{C,j}\varphi \left(t\right)h_{j-1},\\ \frac{\partial E}{\partial W_{ox}}=\frac{\partial E}{\partial {\text{net}}_{o,t}}\frac{\partial {\text{net}}_{o,t}}{\partial W_{ox}}=\delta_{o,t}\varphi \left(t\right)x_{t},\\ \frac{\partial E}{\partial W_{oh}}=\overset{t_{n}}{\underset{j=t_{0}}{\sum }}\delta_{o,j}\varphi \left(t\right)h_{j-1},\\ \frac{\partial E}{\partial b_{f}}=\overset{t_{n}}{\underset{j=t_{0}}{\sum }}\delta_{f,j}\varphi \left(t\right),\\ \frac{\partial E}{\partial b_{i}}=\overset{t_{n}}{\underset{j=t_{0}}{\sum }}\delta_{i,j}\varphi \left(t\right)\frac{\partial E}{\partial b_{C}}=\overset{t_{n}}{\underset{j=t_{0}}{\sum }}\delta_{C,j}\varphi \left(t\right),\\ \frac{\partial E}{\partial b_{o}}=\overset{t_{n}}{\underset{j=t_{0}}{\sum }}\delta_{o,j}\varphi \left(t\right), \end{array}$$

where net_*f*,*t*_, net_*i*,*t*_, net_*C*,*t*_, net_*o*,*t*_ denotes the input of the corresponding function, *δ*_*f*,*t*_=(∂*E*/∂net_*f*,*t*_), *δ*_*i*,*t*_=(∂*E*/∂net_*i*,*t*_), *δ*_*C*,*t*_=(∂*E*/∂net_*C*,*t*_), and *δ*_*o*,*t*_=(∂*E*/∂net_*o*,*t*_).

**Figure 4:**
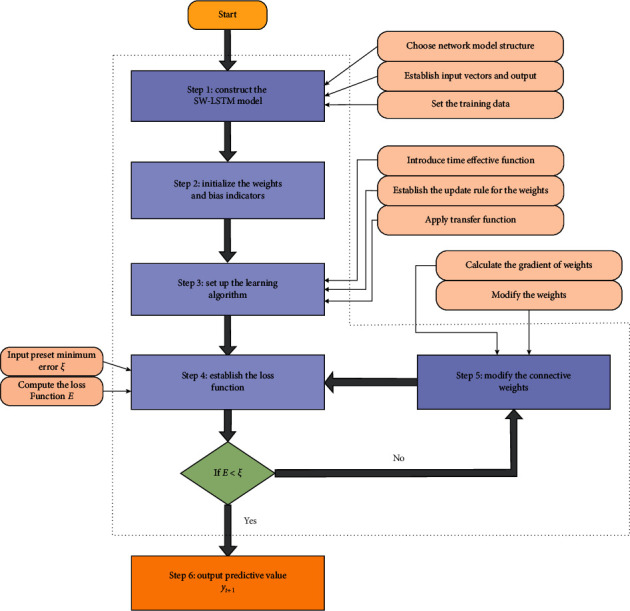
The training algorithm procedures of SW-LSTM model.

The above is the algorithm of SW-LSTM model, which corrects the model parameters accords with the gradient descent method.  Figure 4 illustrates the training algorithm procedures of the proposed model, which involve six steps. For the different subseries of different crude oil series, different hyperparameters, which include the training steps, the number of hidden layers units, the learning rate, number of iterations, and the batch size, should be trained by the proposed model. The specific modelling and empirical prediction are given in Section 4.

### 3.4. Forecasting Process of the Hybrid WPD-SW-LSTM Model

**Figure 5:**
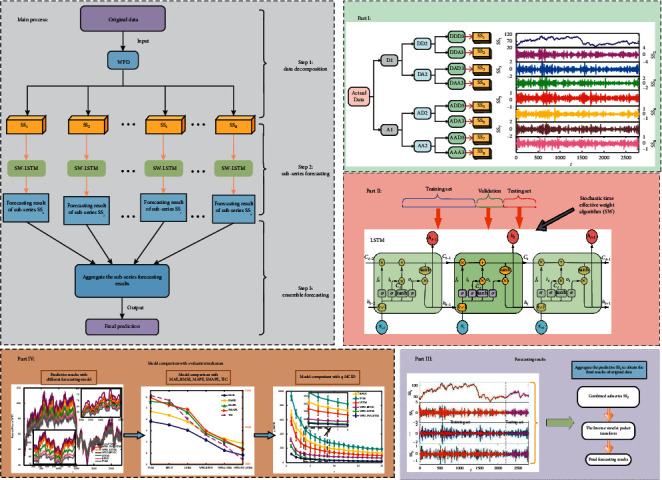
Flowchart of the hybrid WPD-SW-LSTM model.

In this study, the fluctuation of energy futures prices is applied to the proposed hybrid forecasting model, WPD-SW-LSTM. The procedure of the WPD-SW-LSTM approach is described in brief subsequently, and the flowchart of this research is shown in Figure 5. Firstly, the main process of the proposed model is displayed on the upper left of Figure 5, which includes three steps. The first step is data decomposition, where the original preprocessed data are decomposed by WPD method. Then, applying the improved SW-LSTM method for subseries forecasting step, the third step is the ensemble forecasting step. Then, the final forecasting results can be obtained by aggregating the subseries forecasting results with inverse wavelet packet transform. The specific description of each step is as follows:

  Step 1: the WPD technique is employed to analyze the original energy futures series *X*(*t*)(*t*=1,2,…, *N*). And, 8 subseries SS_*i*_,  *i*=1,2,…, 8 are derived from the three-layer WPD method, which indicate that the local oscillations in different frequency bands. The details of the WPD algorithm are given in Section 3.1.
  Step 2: each subsequence SS_*i*_ derived from WPD method is separated into training and testing datasets. The SW-LSTM network is utilized to train and establish the forecasting model on the basis of the training dataset. Model parameters need to be set in advance, which includes the learning rate, the number of hidden layer units, the number of iterations, and the batch size. They are essential for predicting precision of the model. The training algorithm procedures of SW-LSTM model are proposed in Sections 3.2 and 3.3.
  Step 3: it composites the prediction of each SS_*i*_ to obtain the final forecasting results by employing the theory of inverse wavelet packet transform. Moreover, linear regression and relative error are applied to investigate the correlation between predictive points and actual values.
  Step 4: multiple evaluation indicators are adopted to estimate the prediction ability of WPD-SW-LSTM, which involves MAE, RMSE, MAPE, SMAPE, and TIC and a novel method multiple multiorder complexity-invariant distance (MMCID) based on information theory. In addition, other models like SVM, BPNN, LSTM, WPD-BPNN, and WPD-LSTM are taken into account for prediction comparison.

## 4. Forecasting and Statistical Analysis

### 4.1. Data Preprocessing

To estimate the performance of the proposed WPD-SW-LSTM forecasting model, the futures prices of WTI crude oil, Brent crude oil, RBOB gasoline, and heating oil are selected.  Table 1 displays the selected data sets of all indices that are from 02/01/2009 to 23/10/2019. Usually, the non-trading days are regarded as frozen such that this research only adopts the data during trading time. To conduct the experiments, nearly eighty percent of the samples from 2009 to 2017 are used to train the model, and the remaining twenty percent of data are used for testing to examine the effectiveness of the proposed model.  Table 1 provides the selection and division of the four selected oil futures indices. Generally, to minimize the influence of noise and finally enhance the accuracy of forecasting, each subseries SS_*i*_ derived from WPD is normalized to the range of [0,1] by the following standardized method [52, 53]:

(17) $$\begin{array}{c} S{\left(t\right)}^{\prime }=\frac{S\left(t\right)-\min S\left(t\right)}{\max S\left(t\right)-\min S\left(t\right)}. \end{array}$$

After that, to acquire the true predictive value and then intuitively compare the numerical results with the actual value, the normalized output variables *S*(*t*) should be reverted to *S*(*t*) as follows:

(18) $$\begin{array}{c} S\left(t\right)=S{\left(t\right)}^{\prime }\left(\max S\left(t\right)-\min S\left(t\right)\right)+\min S\left(t\right). \end{array}$$

### 4.2. Training and Forecasting by the Hybrid WPD-SW-LSTM Model

In this section, four different energy futures price series are carried out to support the proposed hybrid WPD-SW-LSTM model. The decomposition merit of WPD makes it exceptional in the extraction of feature sequences. The model parameters are trained by calculating the root mean square error between the predicted value and actual value. The global error between the predicted value and the actual target is reduced through weights modification. The training enters the next step when the global error is less than the preset value. For all prediction models involved in this article, the input units are set to 4, and the output units are set to 1. In WPD-SW-LSTM model, the batch size is set to 32, the hidden size is 30, and the epochs number is 400.

**Figure 6:**
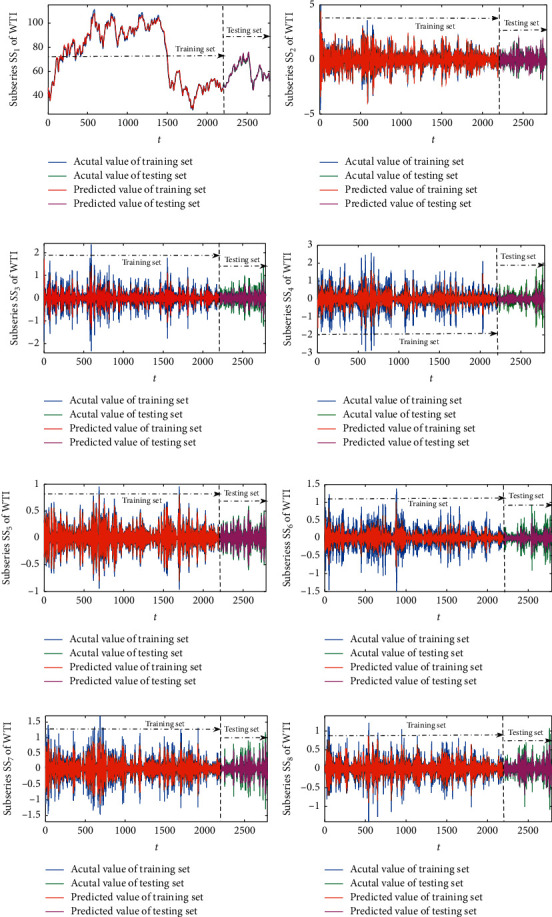
The predicted data and the actual data of each subseries from WTI crude oil. (a) SS_1_. (b) SS_2_. (c) SS_3_. (d) SS_4_. (e) SS_5_. (f) SS_6_. (g) SS_7_. (h) SS_8_.

**Figure 7:**
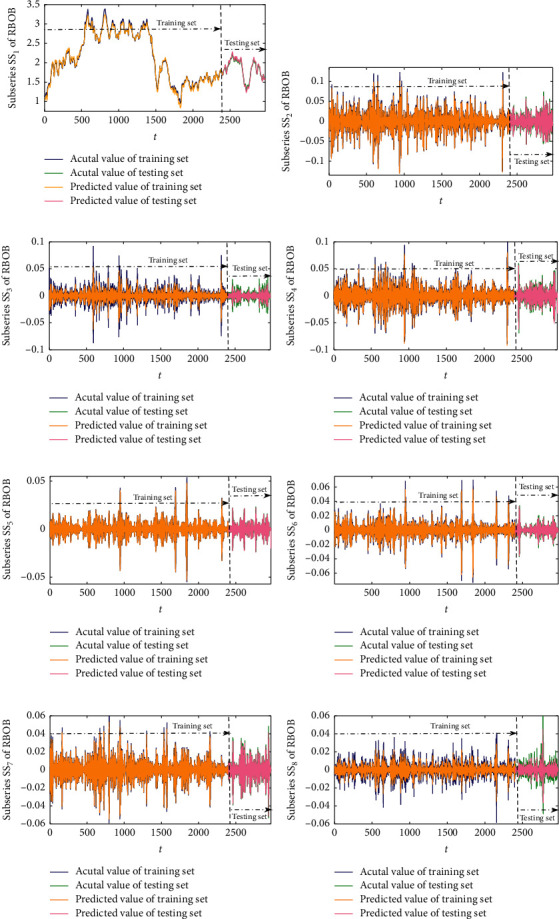
The predicted data and the actual data of each subseries from RBOB gasoline. (a) SS_1_. (b) SS_2_. (c) SS_3_. (d) SS_4_. (e) SS_5_. (f) SS_6_. (g) SS_7_. (h) SS_8_.

Afterwards, the normalized subseries SS_*i*_ obtained from WPD are trained and predicted by the SW-LSTM model. The number of input samples is set to 4, and the number of outputs is set to 1; that is, the 4th order historical data are used to predict the data of the next period.  Figure 6 shows the forecasting results of each subseries from the futures series of WTI crude oil. It is shown visually that the predicted value of each subseries SS_*i*_ is almost consistent with the actual values. With the purpose of illustrating the prediction from the SW-LSTM forecasting model, Figure 7 demonstrates the empirical results of each subseries from RBOB gasoline. Figures 6 and 7 present decomposed forecasting results of WTI crude oil and RBOB gasoline as examples, which is a critical component that measures the fluctuations of the prediction, especially in forecasting the direction of fluctuations accurately. The subseries SS_*i*_ has been recognized as the whole trend of the futures price series, whose results from the proposed forecasting model are well predicted. The curves of the actual data and the predicted data intuitively are very approximating. Then, the final predictive results of the four sample datasets can be calculated by employing the theory of inverse wavelet packet transform.

**Figure 8:**
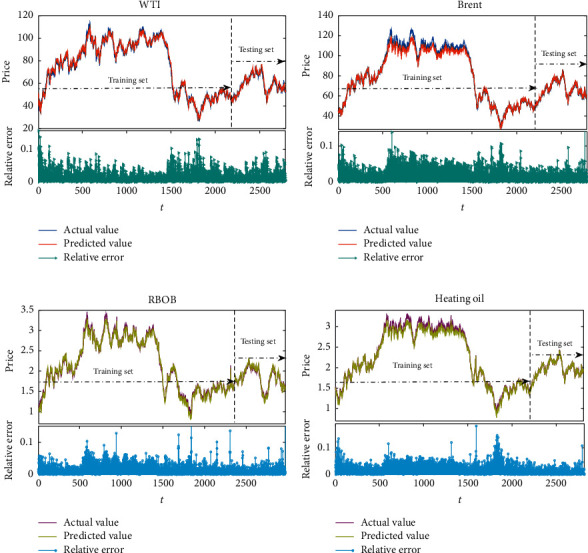
The predicted results of the proposed model for the original series. (a) WTI. (b) Brent. (c) RBOB. (d) Heating oil.

Figure 8 shows the final predictive results for four indices, WTI, Brent, heating oil, and RBOB, with the proposed WPD-SW-LSTM model. From this figure, the fluctuation trends of the predictive data are extremely near that of the actual data. In addition, the absolute correlation error results of the empirical analysis are also revealed in Figure 7, which can be calculated by $\text{RE}\left(t\right)=\left|{\hat{y}}_{t}-y_{t}\right|/y_{t}$. It can be concluded that the predicted results nearly have consistent trends with the fluctuations of the actual data. The results of RE are also centralized in (0,0.01), and only a few sectional data points surpass 0.01 and are smaller than 0.015. It means that with repeated experiments, the energy futures series have been trained excellently, and the forecasting performance of the WPD-SW-LSTM model is improving.

**Figure 9:**
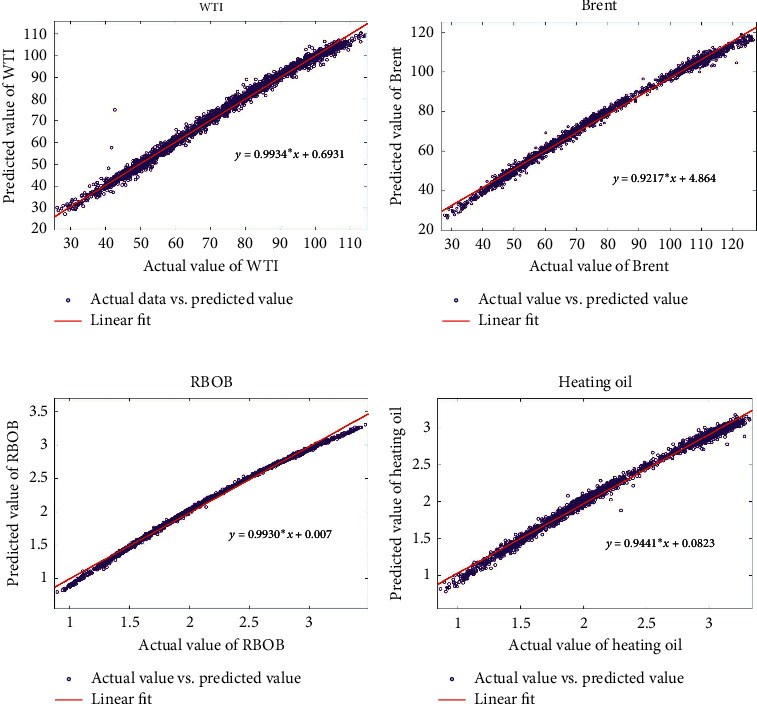
Comparisons of the predicted data and the actual data for the forecasting models. (a) WTI. (b) Brent. (c) RBOB. (d) Heating oil.

It is generally known that the predicted results and the actual value can be fitted by linear regression method, where the predicted points are regarded as the dependent variable *Y*, and the actual data are considered as the independent variable *X*. Through linear regression analysis between the predicted value of the WPD-SW-LSTM model and the actual data, the prediction accuracy can be judged by the goodness of fit. The closer the goodness of fit value is to 1, the closer the predicted value is to the true value. An effective numerical indicator between the two variables is the correlation coefficient *R*. The curves of linear regression for series WTI, Brent, heating oil, and RBOB are revealed, respectively, in Figure 9, and the numerical results are revealed in Table 2. In detail, the values of *R* for these four series are all above 0.98, and the regression coefficients *a* of the linear equations are near to 1, which indicates that the predicted values are almost close to the actual values. The regression equation parameters of the proposed model for WTI are *a*=0.9934, *b*=0.6931, which is approaching to the ideal situation *y*=*x*, followed by the Brent indices, *a*=0.9217, *b*=4.864. The heating oil is *a*=0.9441, *b*=0.0823 and RBOB gasoline is *a*=0.9930, *b*=0.0007.

## 5. Models Comparison and Prediction Accuracy Evaluation

### 5.1. Performance Evaluation Criteria

While the established model WPD-SW-LSTM is utilized to the forecasting experiments, it is also indispensable to validate the forecasting effects of different models. Then, five models (SVM, BPNN, LSTM, WPD-BPNN, and WPD-LSTM) are employed to the forecasting evaluations in this part. Support vector machine (SVM) technique is displayed in this part, which is regarded as the state-of-the-art machine learning theory for binary classification [54–56]. Additionally, to fully prove the effectiveness of the proposed model, BPNN, LSTM, and WPD-BPNN are selected to make a comparison because the proposed model is constructed based on LSTM network, and backpropagation neural network (BPNN) is the most typical neural network. For the purpose of estimating the forecasting error of the new hybrid model and comparing it with other five models, the error measurement between actual data points and predicted value for different models are investigated. Among them, mean absolute error (MAE), root mean square error (RMSE), mean absolute percent error (MAPE), symmetric mean absolute percent error (SMAPE), and Theil inequality coefficient (TIC) are selected as the error evaluation criteria, which can indicate the forecasting performance of each model. Generally, the smaller the error (MAE, RMSE, MAPE, SMAPE, and TIC) values are, the more accurate the predictive ability of the forecasting model is [52]. The evaluation definitions are expressed as follows:

(19) $$\begin{array}{c} \text{MAE}=\frac{1}{N}\sum_{t=1}^{N}\left|y_{t}-{\hat{y}}_{t}\right|,\\ \text{RMSE}=\sqrt{\frac{1}{N}\sum_{t=1}^{N}{\left(y_{t}-{\hat{y}}_{t}\right)}^{2}},\\ \text{MAPE}=100\times \frac{1}{N}\sum_{t=1}^{N}\left|\frac{y_{t}-{\hat{y}}_{t}}{y_{t}}\right|,\\ \text{SMAPE}=100\times \frac{2}{N}\sum_{t=1}^{N}\frac{\left|y_{t}-{\hat{y}}_{t}\right|}{\left|y_{t}\right|+\left|{\hat{y}}_{t}\right|},\\ \text{TIC}=\frac{\sqrt{1/N\sum_{t=1}^{N}{\left(y_{t}-{\hat{y}}_{t}\right)}^{2}}}{\sqrt{1/N\sum_{t=1}^{N}y_{t}^{2}}+\sqrt{1/N\sum_{t=1}^{N}{\hat{y}}_{t}^{2}}}, \end{array}$$

where *y*_*t*_ and ${\hat{y}}_{t}$ are the actual value and the predicted value at time *t*, respectively, and *N* is the total number of the data.

**Figure 10:**
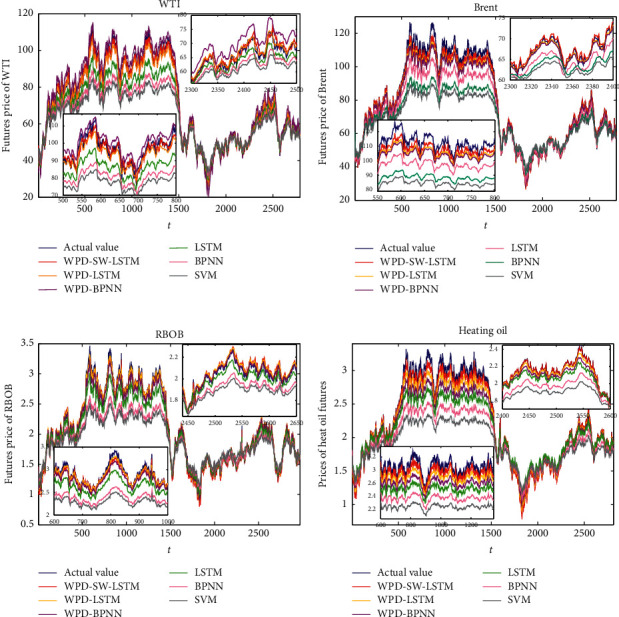
Forecasting comparison of different models for WTI, Brent, heating oil, and RBOB. (a) WTI. (b) Brent. (c) RBOB. (d) Heating oil.

Figure 10 illustrates the forecasting results of WTI, Brent, RBOB, and heating oil for the six forecasting models in comparison. Additionally, the forecasting results from the insert plots of Figure 10 show the local prediction of training sets and testing sets from the proposed WPD-SW-LSTM model, respectively. It displays the distinct advantages contrast with the other five models, SVM, BPNN, LSTM, WPD-BPNN, and WPD-LSTM, especially at big fluctuation stages. Affected by the changes of social economy and various external environment, the energy market shows different fluctuations. Besides, the predicted results during the small fluctuation period seem comparatively accurate for all predictive models.

**Figure 11:**
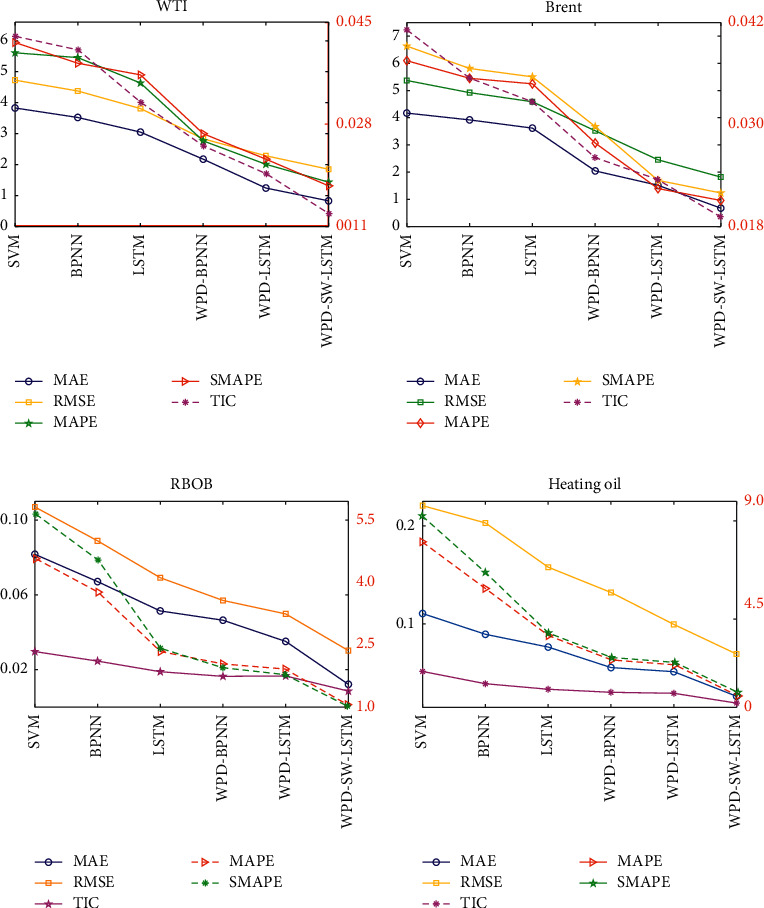
Forecasting comparison of the evaluation errors from the six involved models. (a) WTI. (b) Brent. (c) RBOB. (d) Heating oil.

Tables 3–6 demonstrate a detailed comparison of the evaluation criteria quantitatively, by applying MAE, RMSE, MAPE, SMAPE, and TIC among aforementioned six models. The numerical results demonstrate that the evaluation indicators from the WPD-SW-LSTM model are all the smallest ones among these models, and the evaluation indicators by the hybrid models are almost less than those by the individual models. For example, the MAPE values for WTI futures indices from the first three hybrid models are 1.4329, 2.0092, and 2.7653, and the individual models MAPE values are 4.6351, 5.4562, and 5.6108, respectively. Overall, the empirical results demonstrate that the WPD-SW-LSTM predictor has higher forecasting accuracy. From the error evaluations, the hybrid models WPD-SW-LSTM, WPD-LSTM, and WPD-BPNN are superior to the LSTM, BPNN, and SVM models. Moreover, compared with the WPD-LSTM and WPD-BPNN model, the superior predictive accuracy of the proposed model WPD-SW-LSTM reflects that the stochastic time effective weights (SW) method can play an important role during forecasting process. In particular, after WPD-LSTM is combined with SW, the hyperparameters are extremely improved, and error indicators MAE, RMSE, MAPE, SMAPE, and TIC are raised by 33.32%, 19.14%, 28.69%, 39.59%, and 48.06%, respectively. In order to show the forecast results more intuitively, Figure 11 displays the evaluation values of MAE, RMSE, MAPE, SMAPE, and TIC for different models, respectively. Due to the different data structures and character of these four indices, the left *y*-axis of Figure 11 in the case of WTI and Brent stands for the value of MAE, RMSE, MAPE, and SMAPE, and the right *y*-axis is the TIC value. But for the case of RBOB and heating oil, the left *y*-axis represents the value of MAE, RMSE, and TIC, and the right *y*-axis is the value of MAPE and SMAPE. From Figure 11, the MAPE and SMAPE have similar numerical results for all the case study. The MAPE, SMAPE, and TIC values of RBOB and heating oil indicate that there is no obvious difference between WPD-LSTM model and the WPD-BPNN model, but in accordance with the results of MAE and RMSE, the former is slightly better than the latter model.

In order to verify whether the proposed model is significantly different from other forecasting models (WPD-LSTM, WPD-BPNN, LSTM, BPNN, and SVM), the nonparametric Wilcoxon signed rank test is applied on two absolute errors by two compared models [57–59]. The corresponding statistical test results of the four indexes are presented in Table 7. The results illustrate that the proposed model has statistical significance among the other models. Besides, in Tables 3–6, the error evaluations of MAE, RMSE, MAPE, SMAPE, and TIC by WPD-SW-LSTM are all smaller than those by other five models for indexes WTI, Brent, RBOB, and heating oil. It can be inferred that the WPD-SW-LSTM model is significant superior to other models for the four indexes.

### 5.2. Evaluation of Multiorder Multiscale CID Analysis (MMCID)

In this section, novel error evaluation methods are proposed to detect the predicted performance. The new analysis method is based on complexity-invariant distance (CID) which generally brings about major improvements in time series classification and clustering accuracy [35]. Complexity invariance makes use of knowledge about complexity discrepancy between two different datasets as a modification factor for the existing distance measurement methods [35, 60]. By improving the CID method, multiorder multiscale complexity invariant distance (MMCID) is derived to evaluate the predictions of the energy futures prices with different forecasting models. In practical application, the complexity is not limited to a single scale. The MMCID measurement considers multiple time scales when validating and quantifying the connection between different futures series. The MMCID measurement can consist of the following two procedures: (i) considering one-dimensional discrete time series: *x*_1_, *x*_2_,…, *x*_*i*_,…, *x*_*N*_, consecutive coarse-grained vector *y*^(*τ*)^ is calculated with the scale parameter *τ*. The specific mathematical expressions are as follows, which refers to [61]

(20) $$\begin{array}{c} y_{j}^{\left(\tau \right)}=\frac{1}{\tau }\sum_{i=\left(j-1\right)\tau +1}^{j\tau }x_{i},\;1\leq j\leq \frac{N}{\tau }. \end{array}$$

Particularly, when *τ*=1, the coarse-grained time series is *y*^(1)^, which is merely the primitive sequence. The length of each coarse-grained time series is equal to the length of primitive series divided by the scale parameter *τ*. (ii) According to the principle of CID, we compute the multiorder value of CID for each coarse-grained time series and then acquire the MMCID method as a function with scale parameter *τ*. Assuming that there are two time series, *R* and *S*, with length *n*,

(21) $$\begin{array}{c} R=r_{1},r_{2},\ldots ,r_{i},\ldots ,r_{n},\\ S=s_{1},s_{2},\ldots ,s_{i},\ldots ,s_{n}. \end{array}$$

The multiorder distance expression is given as

(22) $$\begin{array}{c} {\text{ED}}^{q}\left(T\right)={\left(\sum_{i=1}^{n}{\left(r_{i}-s_{i}\right)}^{q}\right)}^{\left(1/q\right)},\\ {\text{CF}}^{q}\left(R,S\right)=\frac{\max\left\{CE^{q}\left(R\right),{\text{CE}}^{q}\left(S\right)\right\}}{\min\left\{CE^{q}\left(R\right),{\text{CE}}^{q}\left(S\right)\right\}},\\ {\text{CE}}^{q}\left(T\right)={\left(\overset{n-1}{\underset{i=1}{\sum }}{\left(t_{i+1}-t_{i}\right)}^{q}\right)}^{\left(1/q\right)},\\ M-\text{CID}\left(R,S\right)={\text{ED}}^{q}\left(R,S\right)\times {\text{CF}}^{q}\left(R,S\right), \end{array}$$

where ED^*q*^(*R*, *S*) between two time series *R* and *S* indicates complexity invariant by introducing a correction index. CF^*q*^ is a complexity correction index, and CE^*q*^(*T*) is a complexity evaluation of time series *T*. Moreover, CF^*q*^ gives reasons for complexity differences of different datasets into comparison. It separates time series with distinctly different complexities to be further apart. And multiorder parameter *q* is applied to enlarge the performance of great changes in the process of error evaluation.

**Figure 12:**
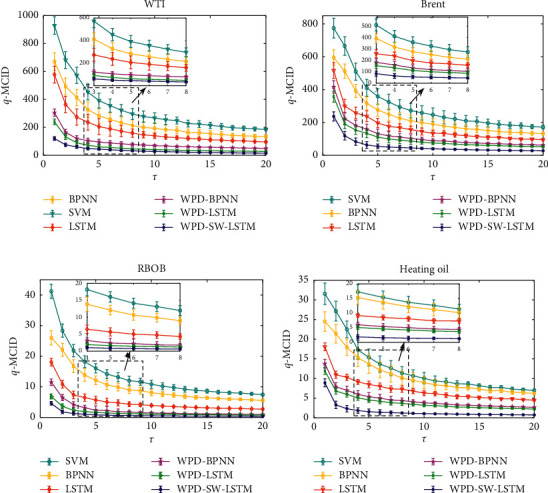
The MMCID curves of the actual futures data points and the forecasting results from different forecasting models. (a) WTI. (b) Brent. (c) RBOB. (d) Heating oil.

When evaluating with the MMCID method, the actual value can be regarded as series *R* and the predicted results as the series *S*. According to the theory of the MMCID, the predicted effectiveness is better when the MMCID value is smaller. It also indicates that the fluctuation trends of the prediction are almost consistent with the actual data. In this study, the parameter *q* is set to 2 and *τ* is from 1 to 20.  Table 8 shows the specific MMCID values between the forecasting results and the actual values from the six mentioned models when the scale parameter *τ*=1. The empirical results from the four different types of experiment data demonstrate that the proposed hybrid model performs much better than the other five forecasting models.  Figure 12 shows MMCID results between the actual futures prices series and the corresponding prediction of them from each predictive model. It is distinctly noticed that the MMCID value between actual data and the prediction ones by the WPD-SW-LSTM model is the smallest one of all, and the results from hybrid models are much better than those from single models for all the four contemplated futures indices. With the novel estimation method, the forecasting merits of the proposed WPD-SW-LSTM model are further manifested, and the productiveness of the SW method added to WPD-LSTM model is also revealed distinctively. In view of the above empirical analysis, the established new hybrid forecasting approach is effective for improving the accuracy of energy futures prices.

### 5.3. Comparative Analysis with Existing Hybrid Models

In this section, the latest hybrid models are considered as the benchmark models to make predictions on the selected four energy futures indexes. Recently, many researchers have combined decomposition methods with machine learning algorithm to establish hybrid forecasting models. Lin et al. [34] proposed the CEEMDAN-LSTM model to the forecast of exchange rate. Niu et al. [32] and He et al. [45] applied the VMD-LSTM model to the forecasting fields of stock prices and exchange rate movements. Li and Wang [62] developed a novel model ST-GRU by embedding stochastic time intensity function into gated recurrent unit model (GRU). Therefore, this section makes comparative analysis between the WPD-SW-LSTM model with the CEEMDAN-LSTM, VMD-LSTM, and ST-GRU models, respectively.  Table 9 has listed the error evaluation results of the four hybrid forecasting models.  Table 10 is the hypothesis test results of Wilcoxon signed rank test for different paired models. The *p* values are all close to 0 and the *H* values are 1 through calculation by hypothesis test, indicating that test rejects null hypothesis. Hence, the prediction error of the WPD-SW-LSTM model is significantly different (under the significance level of 0.05) from the error of the other three hybrid models. Furthermore, compared with the results of other models, all the error evaluations of the forecasting performances in Table 9 are very close, but those of the proposed model are smaller than the errors of the other models. Combined with the results of the statistical test in Table 10, it can be deduced that the prediction efficiency of the proposed model is more superior to the latest three hybrid models for energy futures prices forecasting.

## 6. Conclusion

In this research, a new hybrid forecasting model, WPD-SW-LSTM, has been set up by integrating the wavelet packet decomposition based on LSTM with stochastic time strength weight function method. After decomposing the primitive futures series into several subseries, each forecasting model for the different subseries SS_*i*_ has been established according to its own frequency band properties. The correlation coefficient values (*R*) from four energy futures series are all above 0.98 and extremely near 1, which implies that the proposed model performs great prediction effect. Furthermore, compared with the empirical results of SVM, BPNN, LSTM, WPD-BPNN, and WPD-LSTM forecasting models, the predicted values and different error evaluation reveal that the proposed WPD-SW-LSTM forecasting model has strong points in upgrading the accuracy of energy futures prices. In addition, according to the evaluation errors of MAE, RMSE, MAPE, SMAPE, and TIC, the hybrid models WPD-SW-LSTM, WPD-LSTM, and WPD-BPNN have better prediction performance than the individual models, LSTM, BPNN, and SVM. The effectiveness of stochastic time strength weight function is the key that the accuracy of the WPD-SW-LSTM model is far more than the other five models. By introducing the novel evaluation error, MMCID method and the forecasting effectiveness of the proposed model are further confirmed. At the last section, compared with the recent hybrid CEEMDAN-LSTM, VMD-LSTM, and ST-GRU models, by Wilcoxon test, the proposed model is significantly different from the forecasting errors of the other three models. Combined with the error evaluation results, it can be referred that the forecasting accuracy of the proposed model is the highest among the other benchmark models for energy futures prices forecasting.

## Figures and Tables

**Table 1:** Data selection.

Index	Data sets	Total number	Training sets	Training number	Testing number
WTI	02/01/2009∼23/10/2019	2794	02/01/2009∼31/08/2017	2230	564
Brent	02/01/2009∼23/10/2019	2791	02/01/2009∼21/08/2017	2230	561
RBOB	02/01/2009∼23/10/2019	2976	02/01/2009∼20/11/2017	2380	570
Heating oil	02/01/2009∼23/10/2019	2821	02/01/2009∼22/08/2017	2250	571

Note: training number means the number in training set; testing number represents the number in testing set.

**Table 2:** Linear regression parameters from WPD-SW-LSTM model.

Parameter	WTI	Brent	RBOB	Heating oil
*a*	0.9934	0.9217	0.9930	0.0.9441
*b*	0.6931	4.864	0.0007	0.0823
*R*	0.9901	0.9845	0.9856	0.9822

**Table 3:** Prediction performance evaluation of distinct prediction models for WTI.

Model	MAE	RMSE	MAPE	SMAPE	TIC
WPD-SW-LSTM	0.8283	1.8493	1.4329	1.3143	0.0130
WPD-LSTM	1.2422	2.2842	2.0092	2.1755	0.0195
WPD-BPNN	2.1742	2.8328	2.7653	2.9991	0.0239
LSTM	3.0488	3.8097	4.6351	4.8980	0.0310
BPNN	3.5219	4.3772	5.4562	5.2742	0.0395
SVM	3.8286	4.7274	5.6108	5.9419	0.0417

**Table 4:** Prediction performance evaluation of distinct prediction models for Brent.

Model	MAE	RMSE	MAPE	SMAPE	TIC
WPD-SW-LSTM	0.6756	1.8180	0.9579	1.2339	0.0191
WPD-LSTM	1.5148	2.4533	1.3950	1.6971	0.0235
WPD-BPNN	2.0444	3.5218	3.0684	3.6750	0.0261
LSTM	3.6183	4.5880	5.2474	5.5041	0.0327
BPNN	3.9204	4.9236	5.4507	5.8135	0.0355
SVM	4.1716	5.3706	6.0980	6.6347	0.0412

**Table 5:** Prediction performance evaluation of distinct prediction models for RBOB.

Model	MAE	RMSE	MAPE	SMAPE	TIC
WPD-SW-LSTM	0.0122	0.0302	1.0350	1.0012	0.0085
WPD-LSTM	0.0351	0.0498	1.8576	1.7286	0.0166
WPD-BPNN	0.0464	0.0570	1.9764	1.8878	0.0164
LSTM	0.0514	0.0692	2.2633	2.3379	0.0189
BPNN	0.0671	0.0889	3.6352	4.3773	0.0246
SVM	0.0817	0.1070	4.4089	5.4389	0.0297

**Table 6:** Prediction performance evaluation of distinct prediction models for heating oil.

Model	MAE	RMSE	MAPE	SMAPE	TIC
WPD-SW-LSTM	0.0212	0.0642	0.4775	0.6298	0.0143
WPD-LSTM	0.0463	0.0945	1.8461	1.9538	0.0241
WPD-BPNN	0.0505	0.1271	2.0593	2.1686	0.0252
LSTM	0.0714	0.1529	3.1326	3.2484	0.0284
BPNN	0.0844	0.1980	5.2090	5.9175	0.0340
SVM	0.1056	0.2156	7.2594	8.4040	0.0465

**Table 7:** Wilcoxon signed rank test for proposed model with different prediction models.

		WTI	Brent	RBOB	Heating oil
WPD-LSTM	*H*	1	1	1	1
	*z* value	−39.1953	−42.0197	−7.7244	−20.9041
	Prob.*p*	3.5050*e*^−^10	3.1244*e*^−^12	1.1234*e*^−^14	4.9117*e*^−^6

WPD-BPNN	*H*	1	1	1	1
	*z* value	−22.2057	−21.8502	−30.8334	−20.6775
	Prob.*p*	3.0267*e*^−^6	4.3975*e*^−^9	9.3655*e*^−^29	5.5209*e*^−^9

LSTM	*H*	1	1	1	1
	*z* value	−45.7889	−36.8169	−26.5620	−18.0050
	Prob.*p*	6.0053*e*^−^19	9.8998*e*^−^27	1.8654*e*^−^15	1.7815*e*^−^7

BPNN	*H*	1	1	1	1
	*z* value	−23.8007	−30.5701	−31.1161	−21.1325
	Prob.*p*	3.7303*e*^−^11	8.5523*e*^−^29	1.4575*e*^−^22	3.9941*e*^−^6

SVM	*H*	1	1	1	1
	*z* value	−8.9625	−37.2304	−33.3299	−21.0766
	Prob.*p*	1.6290*e*^−^27	2.1975*e*^−^33	1.4235*e*^−^23	1.3043*e*^−^8

**Table 8:** MMCID value between the actual data and the corresponding predictions.

Index	WTI	Brent	RBOB	Heating oil
WPD-SW-LSTM	120.1289	237.8508	4.5909	8.9039
WPD-LSTM	239.3461	384.7985	6.8226	11.9508
WPD-BPNN	305.2824	416.4688	11.4907	13.7667
LSTM	577.9065	516.0784	18.0672	18.0709
BPNN	730.7260	595.6528	26.0103	24.4857
SVM	929.2038	779.0730	41.2264	31.5295

**Table 9:** Prediction performance evaluation of hybrid forecasting models.

Errors	MAE	RMSE	MAPE	SMAPE	TIC
Index	WTI				
CEEMDAN-LSTM	1.2017	2.1849	1.7099	1.8371	0.0167
VMD-LSTM	1.3158	2.5268	2.2632	2.4196	0.0192
ST-GRU	1.1701	2.2165	1.6895	1.9726	0.0151
WPD-SW-LSTM	0.8283	1.8493	1.4329	1.3143	0.0130

Index	Brent				
CEEMDAN-LSTM	0.9782	2.3274	1.3412	1.5177	0.0228
VMD-LSTM	1.2814	2.5638	1.4101	1.7065	0.0239
ST-GRU	1.1267	2.2680	1.2826	1.4371	0.0215
WPD-SW-LSTM	0.6756	1.8180	0.9579	1.2339	0.0191

Index	RBOB				
CEEMDAN-LSTM	0.0269	0.0381	1.6774	1.5205	0.0168
VMD-LSTM	0.0368	0.0558	1.9783	1.8152	0.0175
ST-GRU	0.0326	0.0328	1.6186	1.4322	0.0116
WPD-SW-LSTM	0.0122	0.0302	1.0350	1.0012	0.0085

Index	Heating oil				
CEEMDAN-LSTM	0.0376	0.0805	1.2376	1.5278	0.0183
VMD-LSTM	0.0497	0.1014	1.8509	2.0125	0.0252
ST-GRU	0.0408	0.0732	1.0338	1.2957	0.0171
WPD-SW-LSTM	0.0212	0.0642	0.4775	0.6298	0.0143

**Table 10:** Wilcoxon signed rank test for proposed model with different hybrid models.

		WTI	Brent	RBOB	Heating oil
CEEMDAN-LSTM	*H*	1	1	1	1
	*z* value	−38.4806	3.4128	23.3774	3.0441
	Prob.*p*	3.1639*e*^−^10	6.4302*e*^−^4	7.2652*e*^−^12	0.0023

VMD-LSTM	*H*	1	1	1	1
	*z* value	−20.6386	7.2933	21.2588	−2.7126
	Prob.*p*	1.2432*e*^−^9	3.0256*e*^−^13	2.7349*e*^−^10	0.0067

ST-GRU	*H*	1	1	1	1
	*z* value	−23.4035	−15.9011	−24.8309	−5.3581
	Prob.*p*	3.9388*e*^−^12	6.2225*e*^−^15	4.1571*e*^−^13	8.4113*e*^−^8

## Data Availability

The data used to support the findings of this study are available from the corresponding author upon reasonable request.
